# Supplementary material for: Reconciled rat and human metabolic networks for comparative toxicogenomics and biomarker predictions
Source: Nat Commun. 2017 Feb 8;8:14250. doi: 10.1038/ncomms14250 (PMC5309818; doi:10.1038/ncomms14250)
Supplement: Supplementary Information — Supplementary figures, supplementary methods and supplementary references. [file ncomms14250-s1.pdf]

Supplementary Figures

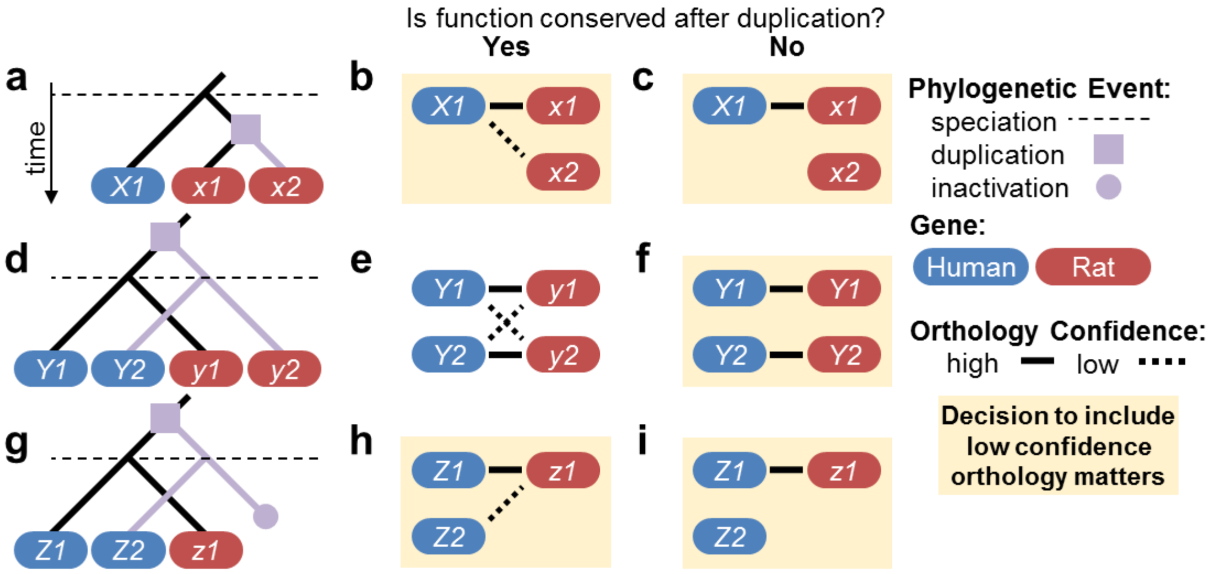

Supplementary Figure 1 – Inferring function between orthologs is not trivial.

(**a**) Example of the evolutionary history of a single gene in the most recent common ancestor of rats and humans that underwent a duplication event in rats but not humans after speciation. From this evolutionary relationship, *X1* may be annotated to two orthologs, *x1* and *x2*. Orthologous pairs of rat and human genes separated by shorter evolutionary distances were classified as high confidence and assigned the same number.

(**b**) Assuming that function is conserved across *X1*, *x1*, and *x2* after speciation and duplication events, metabolic reactions associated with *X1* in a human GENRE should be associated with *x1* and *x2* as isozymes in a rat GENRE. This example highlights the importance of including multiple orthology annotations when converting GPR rules between species, even when *X1* and *x1* has stronger evidence for orthology than *X1* and *x2*.

(**c**) Assuming *X1* and *x1* catalyze the same metabolic function but *x2* evolved an affinity for a different substrate after duplication, metabolic reactions associated with *X1* in a human GENRE should only be associated with *x1* and not *x2*. This example suggests that some orthology annotations may need to be discarded during the GPR conversion process (and potentially

assigned to a new rat-specific reaction).

(**d**) Evolutionary history of a single ancestral gene that was duplicated before speciation resulting in two human genes, *Y1* and *Y2*, and two rat genes, *y1* and *y2*.

(**e**) Assuming that function is conserved across *Y1*, *y1*, *Y2*, and *y2* after duplication and speciation events, metabolic reactions associated with *Y1* and *Y2* as isozymes in a human GENRE should be also be associated with *y1* and *y2* as isozymes in a rat GENRE.

(**f**) If the ancestral gene of *Y2*/*y2* evolved a novel function shortly before speciation and after duplication from the ancestral gene of *Y1*/*y1*, integrating low confidence orthology annotations between *Y1*/*y2* and *Y2*/*y1* into the GPR conversion process could generate GPR rules with twice as many rat genes as human genes.

(**g**) Evolutionary history of a single ancestral gene that was duplicated before speciation resulting in two human genes, *Z1* and *Z2*, but only one rat gene, *z1*, after a loss of function mutation in the rat descendent of *Z2*'s ancestral gene.

(**h**) Assuming that function is conserved between *Z1*, *z1*, and *Z2*, metabolic reactions associated with *Z1* and *Z2* as isozymes in a human GENRE would only be catalyzed by *z1* in a rat GENRE.

(**i**) If *Z1* and *Z2* were known to catalyze distinct reactions in a human GENRE, low confidence orthology annotations between *Z2*/*z1* might inappropriately suggest the addition of a human-specific reaction to a rat GENRE.

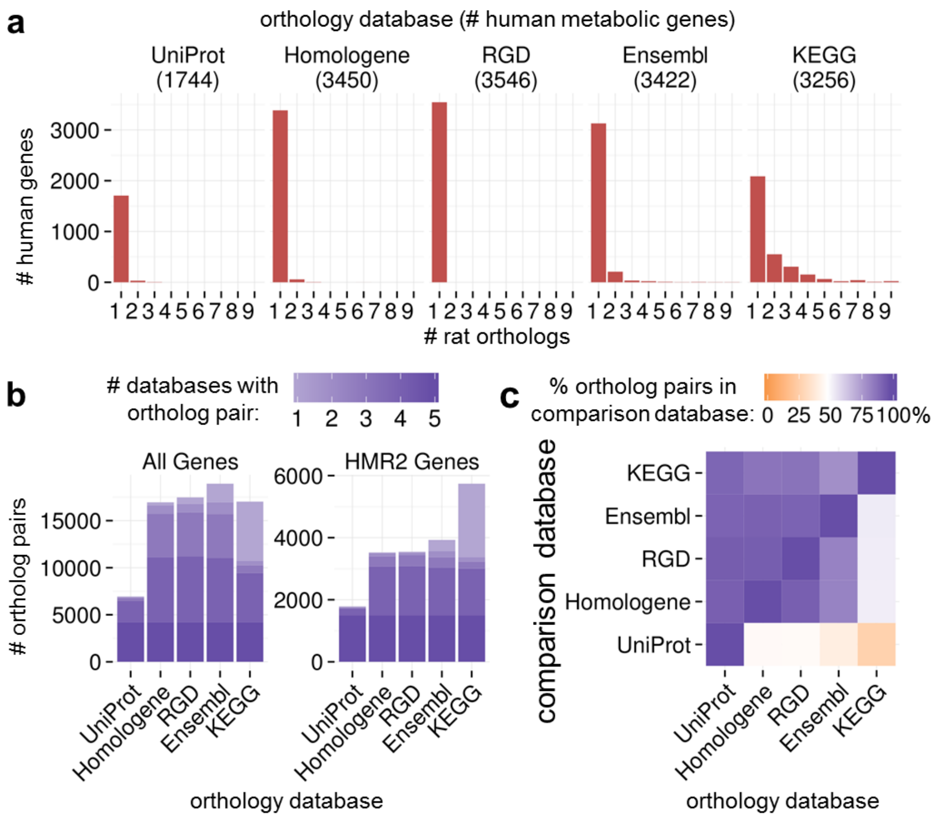

Supplementary Figure 2 – Summary of orthology annotations between rat and human genes from five orthology databases.

(**a**) Distributions of the numbers of rat orthologs annotated to individual human genes from each database. Numbers below each database name indicate the total numbers of human metabolic genes from HMR2 with at least one rat ortholog. Human genes with more than 9 orthologs are not shown.

(**b**) Numbers of ortholog pairs from each orthology database that are also annotated in other orthology databases. Lighter and darker purple represent weaker and stronger consensus among databases, respectively.

(**c**) Percent of ortholog pairs in each database (x-axis) that overlapped with in orthology annotations in other databases (y-axis).

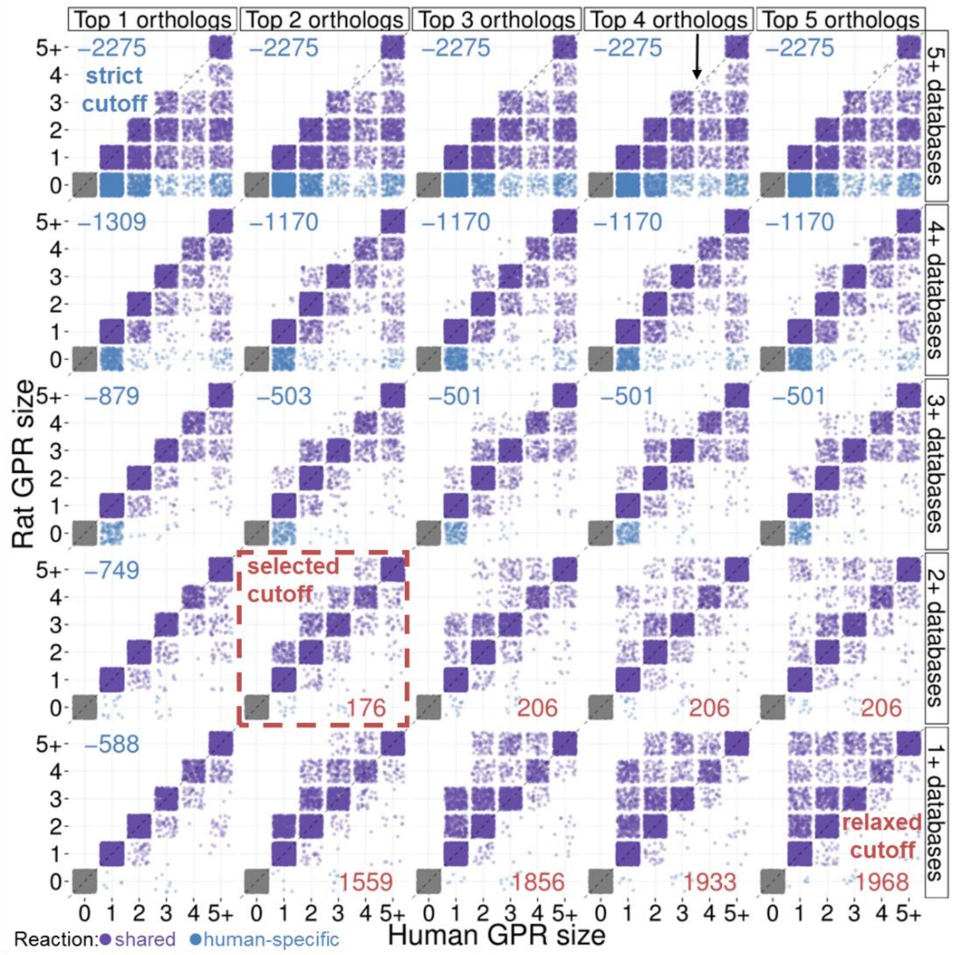

Supplementary Figure 3 – Converting GPR rules using a consensus approach.

Using a consensus approach, a subset of high-quality orthology annotation from 5 databases was obtained to generate an automated draft of a rat metabolic network based on a human metabolic network. Sensitivity analysis of two parameters used to filter orthology annotations on the relative sizes of rat and human GPR rules: minimum database count (by row) and maximum orthology rank (by column). Smaller orthology rank thresholds limited fewer rat orthologs to be replaced by individual human genes. Larger database count thresholds removed ortholog pairs that were not annotated frequently across multiple databases. Dots represent the relative rat and human GPR sizes for individual reactions and the number highlighted in each panel represents the number of shared reactions with larger rat than human GPR sizes minus the number of shared reactions with larger human than rat GPR sizes. Using orthology annotations

from any database (bottom row) generated rat GPR rules that were frequently larger than the original human GPR rules, unless each human gene was limited to one ortholog (bottom left panel). Requiring orthology annotations to be described by at least 3 different databases (middle row) increased the numbers of reactions automatically annotated as human-specific (blue dots) and provided human GPR rules that were frequently larger than rat GPR rules. Ultimately, the pair of selected cutoff parameters (boxed panel) used in the GPR conversion process provided balanced numbers of disproportionately sized GPR rules between draft rat and human networks.

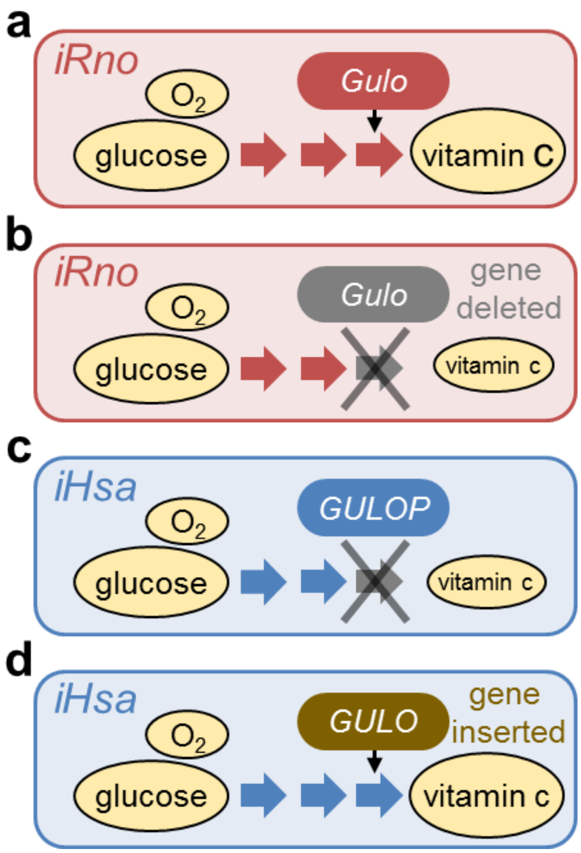

Supplementary Figure 4 – Simplified metabolic network diagrams capturing the known functional importance of L-gulonolactone oxidase in vitamin C synthesis.

(**a**) Rats are capable of synthesizing vitamin C from limited substrates which was captured by *iRno*. The last enzymatic step of this process is known to be catalyzed by *Gulo*.

(**b**) By simulating the deletion of *Gulo* with *iRno*, rats were no longer predicted to be capable of synthesizing vitamin C given glucose and oxygen.

(**c**) Humans cannot synthesize vitamin C from limited substrates which was captured by *iHsa*. The human ortholog of the rat gene, *Gulo*, is a non-functional pseudogene.

(**d**) By simulating the knock-in of a functional equivalent to *Gulo* in humans, *iHsa* was capable of performing *de novo* vitamin C synthesis.

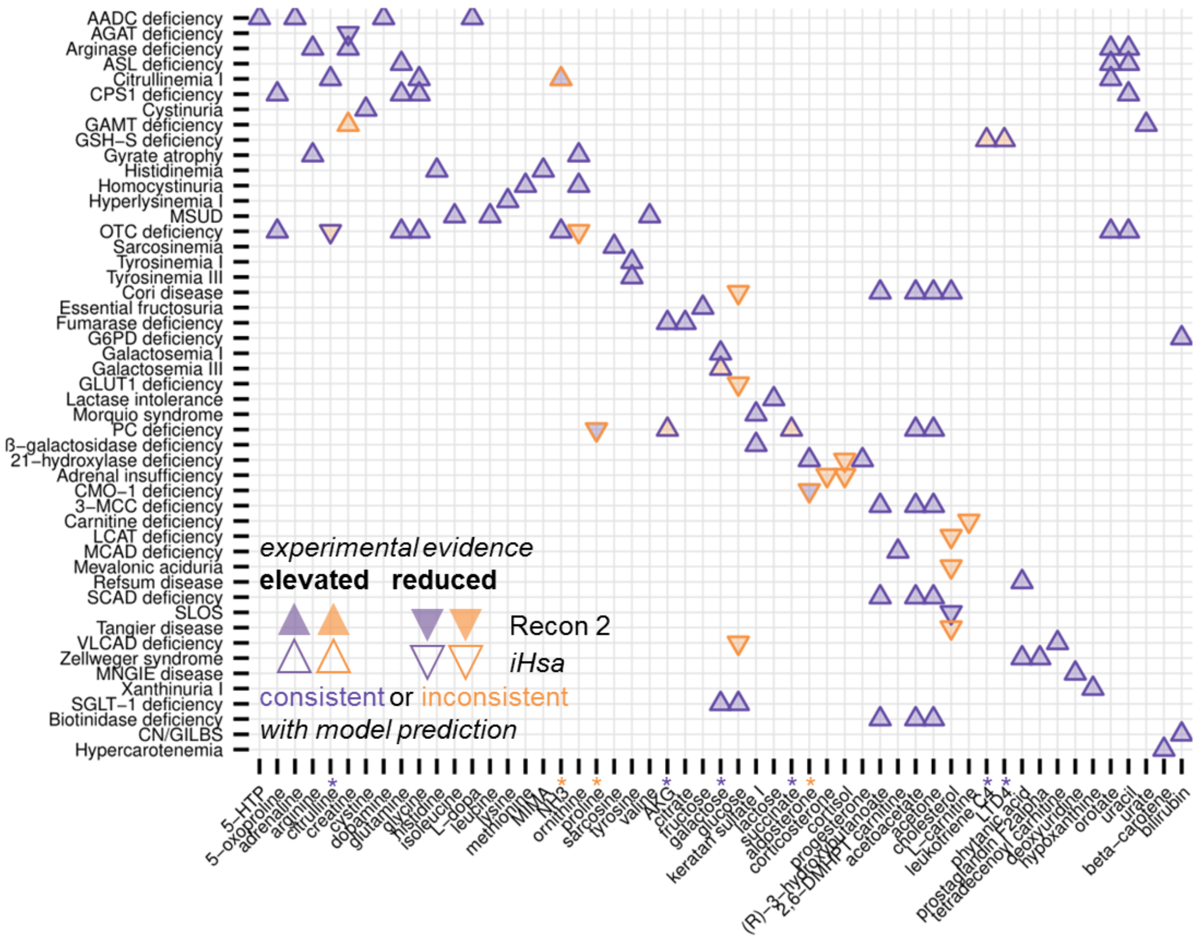

Supplementary Figure 5 – Biomarker predictions for inborn errors of metabolism (IEMs)

Comparisons between biomarker predictions generated by *iHsa* and *Homo sapiens* Recon 2[1] against known metabolite biomarkers for IEMs [1,2]. Triangles pointing up and down represent biomarkers known to be elevated or reduced in patients with IEMs. Purple and orange colors represent predictions that were either consistent or inconsistent, respectively, with *iHsa* (triangle outline) and/or Recon 2.04 (triangle filling). Biomarker predictions with increased (purple asterisks) or decreased (orange asterisks) performance in *iHsa* compared to Recon 2 (version 2.04) are highlighted for individual metabolites. Metabolites abbreviations: methyl-imidazole acetic acid (MIMA); 5−hydroxy−L−tryptophan (5-HTP); 2,6−dimethylheptanoyl−carnitine (2,6-DMHPT-crn).

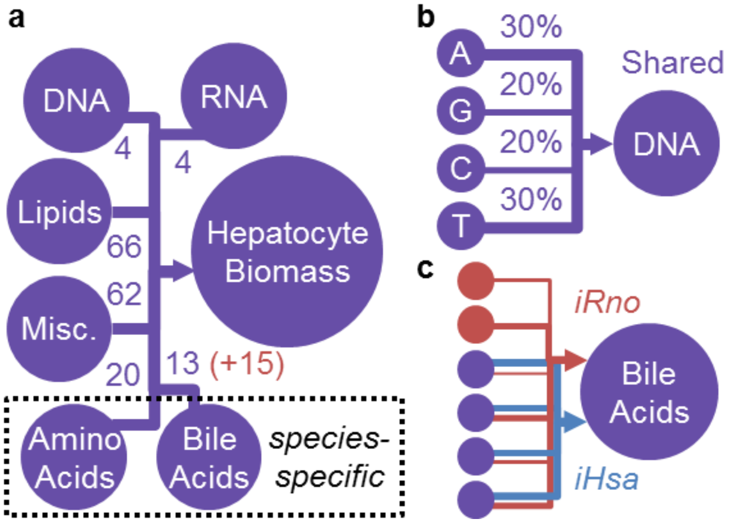

Supplementary Figure 6 – A unified biomass reaction was created for rat and human hepatocytes to enable comparative predictions using *iRno* and *iHsa*.

(**a**) Each metabolite consumed in the hepatocyte biomass reaction represents an "average" biomass subcomponent that is synthesized in a separate reaction (**b**-**c**). DNA, RNA, lipids, and miscellaneous metabolites (misc) like glycogen and vitamin C were assigned species-independent synthesis reactions. Bile acids and amino acids, which can vary significantly between species, were assigned species-specific synthesis reactions in *iRno* and *iHsa*. Numbers indicate how many unique metabolites are shared (purple) or rat-specific (red) within each biomass subcomponent.

(**b**) The biomass subcomponent for an average DNA molecule is produced by consuming experimentally-derived ratios of individual deoxynucleotides. In this reaction, more adenine (A) and thymine (T) are incorporated into DNA than cytosine (C) and guanine (G) as indicated by percent labels and by line thickness.

(**c**) Synthesis of an "average" bile acid was defined separately for *iRno* and *iHsa* in order to account for species-specific metabolites (muricholic acids) and relative abundances.

*Each lipid metabolite is comprised of glycerol backbones with 1-3 fatty acid chains of various lengths, representing over 100 unique metabolites, as extensively curated in HMR2.

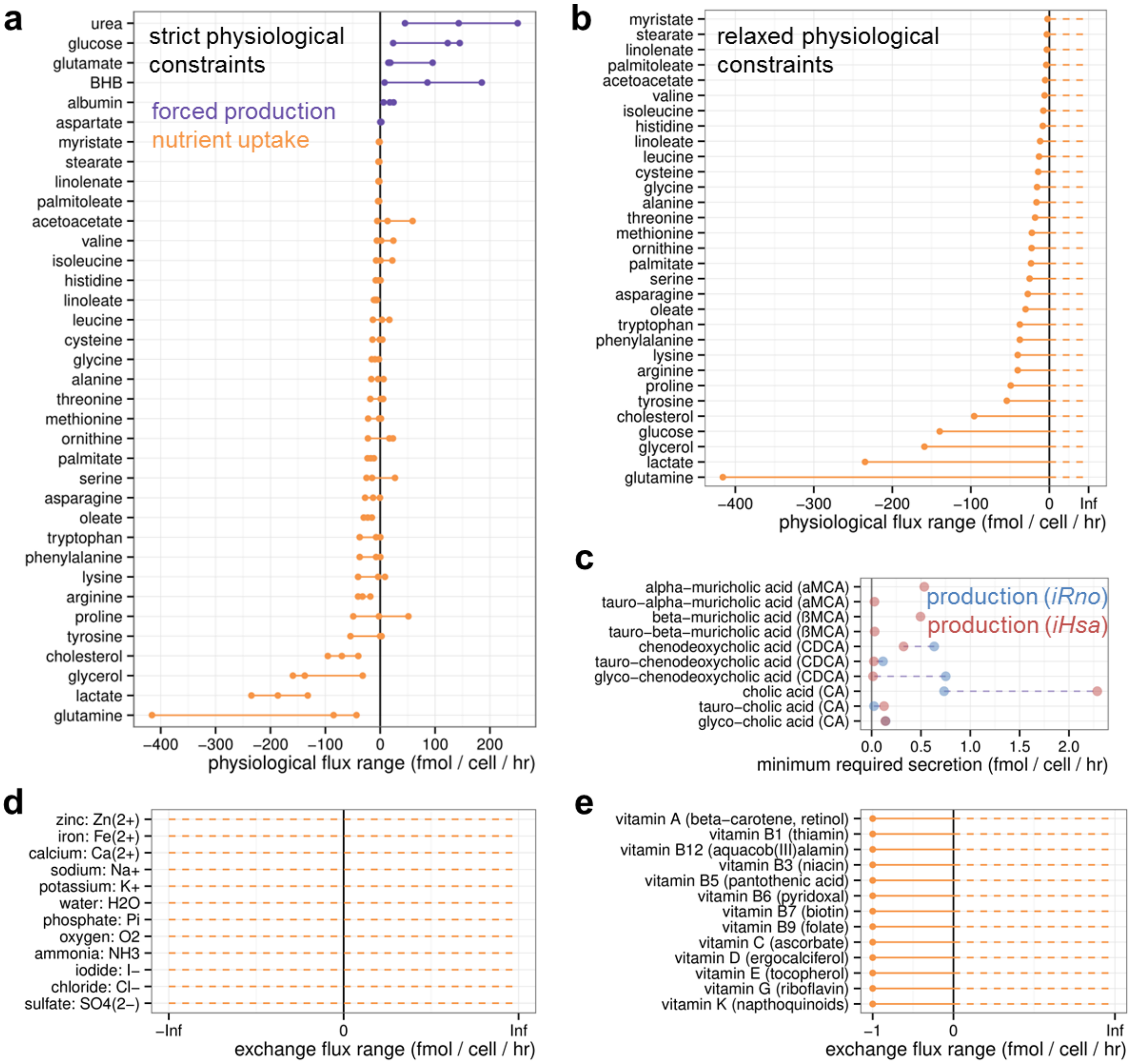

Supplementary Figure 7 – Physiological constraints applied to *iRno* and *iHsa*.

(**a**) Experimentally reported flux measurements from rat hepatocytes were obtained from six separate studies to constrain *iRno* and *iHsa* with exchange boundaries that represent physiological conditions. Minimum, median, and maximum reported exchange fluxes in fmol cell^{-1} hour^{-1} are shown for each metabolite. Reaction lower bounds for exchange metabolites were set to the minimum reported value (leftmost point) as strict physiological constraints. For simulations of hepatocyte growth using the biomass objective, the maximum value for each metabolite was also applied as the upper bound to exchange reactions. Exchange fluxes for

albumin were scaled to represent the secretion of an average amino acid from albumin because the albumin metabolite represents a full-length protein with 608 amino acids (in rats).

(**b**) Experimentally reported flux measurements from (**a**) were also applied as relaxed physiological constraints for toxicogenomics biomarker predictions. Reaction lower bounds with positive values (forced production) to were set to zero and upper bounds with negative values (forced consumption) were set to positive infinity (10^6).

(**c**) Species-specific constraints required distinct quantities of bile acids to be produced under strict physiological conditions in hepatocytes. Each point represents the lower bound applied to either *iRno* (red) or *iHsa* (blue) based on serum concentrations in rats and humans, respectively. Synthesis and secretion of α**-** and β**-**muricholic acids in both taurine-conjugated and unconjugated forms were only requirements for *iRno*.

(**d**) Inorganic ions were allowed unconstrained consumption rates of -10^6 fmol $cell^{-1}$ $hour^{-1}$.

(**e**) Cofactors and vitamins considered essential in humans were set to an uptake value of 1 fmol $cell^{-1}$ $hour^{-1}$ in rat and human networks.

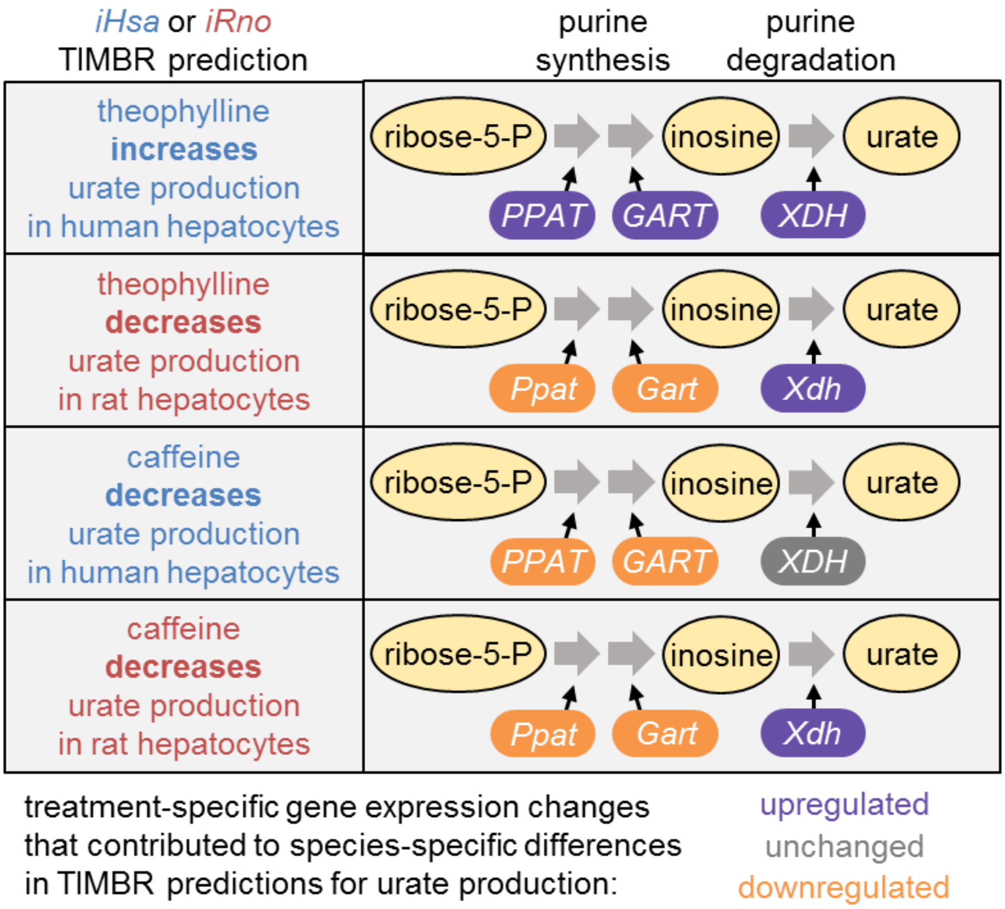

Supplementary Figure 8 – By comparing reaction weights and reaction fluxes associated with urate production, we found that two human enzymes involved in *de novo* purine synthesis, *PPAT* and *GART*, were upregulated in response to theophylline but downregulated in response to caffeine. In contrast, rat orthologs for these genes (*Ppat* and *Gart*) were downregulated by both caffeine and theophylline. A human enzyme involved in purine degradation, xanthine oxidase (*XDH*), was also upregulated by theophylline but unaffected by caffeine; however, the rat ortholog, *Xdh*, was upregulated in response to both compounds. These results suggest that activation of purine synthesis and purine degradation pathways might also play mechanistic roles in the human-specific elevation of urate after theophylline treatment.

Supplementary Methods

Converting a human metabolic network into a draft rat metabolic network

The first step in transforming a human metabolic model into a rat metabolic model involved assigning GPR rules consisting of rat genes to reactions in HMR2[3] associated with human GPR rules. Metabolic networks are typically comprised of two fundamental components: the stoichiometric relationships between metabolites and reactions, termed the stoichiometric matrix or S-matrix, and Boolean relationships between enzymes that catalyze a reaction, termed gene-protein-reaction GPR association rules. Reactions and metabolites in the S-matrix were assumed to be organism-independent because the molecular building blocks of a cell such as amino acids, nucleic acids, and membrane lipids are generally consistent across multiple species. In contrast, enzymes catalyzing reactions within the GPR rules of HMR2 were specifically encoded by the human genome, necessitating distinct formulations of GPR rules for the rat genome.

Inferring metabolic function through orthology annotations

To construct a genome-scale network reconstruction (GENRE) of rat metabolism, human genes assigned to metabolic reactions in a human GENRE through gene-protein-reaction (GPR) relationship rules were replaced with orthologous rat genes. Inferring metabolic function through orthology is not trivial because orthologs descended from the most recent common ancestor of rats and humans have endured more than 50 million years of evolutionary pressures (**Supplementary Fig. 1**). Additionally, mutations involving the duplication and/or inactivation of gene after speciation can lead to one-to-many, many-to-many, or many-to-one orthology annotations between rat and human genes[4].

A consensus approach was used to assign a confidence score to each pair of human and rat orthologs for initial construction of the rat GENRE, *iRno*. We developed a quantitative method to infer metabolic function by incorporating the collective efforts of multiple genome annotation communities: UniProt, Homologene, RGD, Ensembl, and KEGG. Using these five orthology databases, we assigned a confidence score to deprioritize the conversion of ortholog pairs that were annotated in fewer databases. This prioritization step was implemented to filter out low confidence orthology annotations due to the possibility that function may not be conserved as described in **Supplementary Fig. 1a**. We anticipate that using orthology annotations derived from multiple computational methods will be more robust than quantitative methods such as BLAST because a single point mutation could be sufficient to alter the basic function of a metabolic enzyme while orthologs with low sequence similarity can catalyze similar reactions[4].

We compared orthology databases to highlight the advantages and disadvantages of using a consensus approach versus an individual database (**Supplementary Fig. 2**). Surprisingly, the distribution of rat orthologs annotated to each human gene varied substantially between orthology databases (**Supplementary Fig. 2a**). Over a third of human genes were annotated to two or more rat orthologs in KEGG while RGD was restricted to one rat ortholog per human gene. Despite this limitation, RGD had the highest coverage of human genes with orthology annotations compared to KEGG which had the second least. UniProt covered the fewest human genes but most orthology annotations were consistently found in at least 4 of 5 orthology databases (**Supplementary Fig. 2b**). Based on this information, orthology annotations from UniProt alone might not be sufficient to carry out the GPR conversion process at the genome-scale; however, UniProt could be useful in a consensus-based GPR conversion method by reinforcing confidence in a core subset of well-annotated ortholog pairs. Most ortholog pairs were annotated in multiple databases although KEGG also included a large number of unique ortholog pairs (**Supplementary Fig. 2b**). Each database added between 12 and 514 unique

human genes originally represented in HMR2 and between 16 and 2371 unique ortholog pairs not found in any of the other four databases. The percentage of non-overlapping ortholog pairs between any two databases was less than 50% with the exception of UniProt (**Supplementary Fig. 2c**). Despite a moderate degree of overlap, these data suggest that no consensus has been established among orthology annotation resources. As an alternative to choosing a single orthology database, a consensus approach would reduce the number of unconverted human genes and potentially capture more evolutionary differences in between rats and humans (**Supplementary Fig. 1**).

Aggregating orthology annotations from multiple databases increases the risk of inappropriately replacing human genes with rat orthologs that do not perform the same function. To identify a high-quality subset of orthology information that preserved functionality and GPR sizes between drafts of *iRno* and *iHsa*, we developed a consensus-based GPR conversion algorithm that required orthologs to be annotated in at least 1-5 databases and limited individual human genes to be replaced by a maximum of 1-5 rat orthologs (**Supplementary Fig. 3**). The orthology database consensus score was defined as the number of databases in which a unique pair of rat and human genes was annotated. Rat and human genes were represented with Entrez gene identifiers to evaluate the presence of ortholog pairs across all five databases. Entrez gene mappings were included by default for RGD, Homologene, and KEGG orthology databases while UniProt protein entries and Ensembl gene identifiers were mapped to Entrez genes by customizing data output options within each database. When limiting the maximum number of rat orthologs that replace each human gene, orthologs were first prioritized by consensus orthology scores followed by gene scores based on genome annotation information from NCBI, UniProt, and Ensembl. Four subjective parameters were discretized into integer values between zero and five and summed to calculate gene scores: UniProt gene evidence level (protein = 5; transcript = 4; inferred from homology = 3; predicted = 2; uncertain = 1; no data = 0), UniProt

Annotation Score (score between 1 and 5 = value; no data = 0), Ensembl gene status (known = 5; known by projection = 4; novel = 3; putative = 1; no data = 0), NCBI protein-coding evidence (evidence = 5; otherwise = 0). Rat orthology ranks for each human gene in each reaction were determined by sorting consensus orthology scores in descending order followed by rat gene score in descending order.

Without filtering orthology annotations, rat GPR rules were often much larger than human GPR rules. After manually checking the accuracy of rat GPR rules in the context of genome annotations, orthology annotations between closely related but functionally distinct enzymes were common after converting the unfiltered set of orthologs. The orthology database score and the orthology rank were developed as filtering metrics to avoid inferring function based on non-specific orthology annotations that can be interpreted as false positives for metabolic reactions. However, accurate orthology annotations would also be discarded when applying stringent threshold values, decreasing the sensitivity of the approach. Instead of choosing arbitrary cutoffs for these parameters, a network-driven approach was developed to identify a subset of orthology annotations that maintains a reasonable balance between sensitivity and specificity.

A network-driven approach was used to select cutoff values for the minimum orthology database score and the maximum orthology rank. We found that converting all orthology annotations present in any of the 5 orthology databases generated rat GPR rules with disproportionately more genes compared to the original human GPR rules (bottom row of panels in **Supplementary Fig. 3**). We assumed that this difference was more likely explained by a large number of false positive orthology annotations than an actual genome-scale difference in the redundancies between rat and human metabolic enzymes. Alternatively, requiring orthologs to be annotated in all 5 databases generated much smaller rat GPR rules for shared reactions and introduced a large number of human-specific reactions that would

substantially reduce the functionality of *iRno* (top row of panels in **Supplementary Fig. 3**). We found a balanced relationship between the numbers of reactions with larger rat GPR rules than human and numbers of reactions with larger human GPR rules than rat by removing orthology annotations found in only one database and restricting the replacement of each human gene to two rat orthologs (selected cutoff panel in **Supplementary Fig. 3**). Additionally, the selected cutoff preserved the same functionalities as the relaxed cutoff when evaluating metabolic tasks from HMR2[3]. This filtering step was important because methods that integrate gene expression data or simulate the impact of genomic alterations rely heavily on the number of redundant enzymes associated with a reaction[5,6].

Identifying species-specific reactions

We initially explored the Kyoto Encyclopedia for Genes and Genomes[7,8] (KEGG) database as a starting point for identifying species-specific differences between rat and human metabolism. Prior to adding potential new reactions to *iRno* and *iHsa*, existing reactions and metabolites were updated with annotations to external databases. Throughout the entire reconstruction process, 742 reactions and 354 metabolites were assigned new or updated KEGG annotations to replace empty, incorrect, generic, or obsolete KEGG identifiers. The numbers of unique KEGG REACTION and KEGG COMPOUND identifiers represented across *iRno* and *iHsa* increased from 1376 and 1650 to 1702 and 1721, respectively, compared to HMR2 (**Supplementary Data 3**).

Updated annotations were necessary to avoid creating duplicate entries of unique reactions or metabolites and to facilitate assigning GPR rules to 122 reactions not previously associated with any genes. For example, the metabolic reaction catalyzing the conversion of threonine to glycine and acetaldehyde was originally present in HMR2 as a spontaneous (non-enzymatic) reaction with no external annotations[3]. This reaction was identified in the KEGG database as the

rat-specific reaction, threonine aldolase (R00751). As a result, this reaction was assigned a new GPR rule in *iRno* and disabled in *iHsa*. Of 18 rat-specific KEGG reaction annotations: 5 were already represented and removed from *iHsa*; 11 were added as new rat-specific reactions *iRno*; and 2 redundant with other rat-specific reactions were ignored (**Supplementary Data 8**). Of 75 human-specific KEGG annotations: 4 were already represented in *iHsa*, and had been disabled in *iRno* as a result of the GPR conversion process; 14 were re-classified as shared reactions after identifying suitable rat orthologs; and 57 involved in peripheral pathways such as xenobiotic metabolism were ignored (**Supplementary Data 8**).

iRno and *iHsa* were expanded and updated in parallel when possible to maintain consistency in the reconstruction process. For each new reaction added, rat and human GPR rules were constructed manually using evidence from experimental literature and functional annotation databases (**Supplementary Data 1**). Evidence supporting the presence of a reaction in one organism and not the other was necessary for classifying a reaction as species-specific. Otherwise, reactions directly associated with rat and human enzymes or indirectly through orthology annotations were assumed to be shared. In total, 69 biochemical, 32 transport, 40 exchange reactions were added. All transport and exchange reactions were shared by *iRno* and *iHsa* and nine biochemical reactions were unique to *iRno*.

iRno and *iHsa* were expanded to include species-specific reactions from the KEGG database and literature sources. Lists of reactions and modules linked to genes annotated in humans (hsa) and rats (rno) were obtained using KEGG's Representational state transfer (REST)-style interface (http://www.kegg.jp/kegg/rest/keggapi.html). Reactions linked to humans and not rats or to rats and not humans were manually investigated for their feasibility as actual species-specific reactions. To identify potential differences between the metabolic capabilities of rats and humans from literature, various searches were performed using PubMed

(http://www.ncbi.nlm.nih.gov/pubmed) with combinations of the keywords: rat, human, comparative genomics, cross-species, species-specific, metabolism, metabolic deficiency. No comprehensive comparative analyses were identified other than original publication of the rat genome[9].

Curating GPR rules to include complex relationships

GPR rules comprised of more than one gene were initially limited to isozymic "or" relationships because none of the 1390 unique GPR rules in HMR2 described relationships between subunits in a protein complex[3]. To overcome this limitation, GPR rules were manually constructed to include "and" logical operators for both rat and human models when possible. Evidence supporting the requirement of multiple enzymatic subunits to perform a metabolic function were obtained from functional annotation databases and experimental literature (**Supplementary Data 1**). We also compared complex human GPR rules from the second largest human GENRE, *Homo sapiens* Recon 2 (versions 2.0.3 and 2.0.4), with an early draft of *iHsa* in order to convert isozymic relationships into complex relationships.

Formatting complex GPR rules for TIMBR

For gene expression integration using TIMBR (Transcriptionally-Inferred Metabolic Biomarker Response), GPR rules involving redundant subunits in a protein complex were structured according to the following format: (*A1* or *A2*) and (*B1* or *B2*), where redundant enzymes are grouped together for each subunit. With this GPR format, a TIMBR weight represents an average change in gene expression for the subunit that experienced the largest perturbation. Because TIMBR summarizes directional changes instead of absolute values, the following alternative Boolean representation could yield different results: (*A1* and *B1*) or (*A1* and *B2*) or (*A2* and *B1*) or (*A2* and *B2*), where non-redundant subunits are grouped together for each possible protein complex. With this alternative representation, a reaction weight would represent

an average of the largest gene expression changes observed for each possible protein complex. Although both approaches are conceptually similar, TIMBR implements the former approach that summarizes gene expression changes independently for each subunit.

Recapitulating biological functions with metabolic tasks

Curated rat and human models successfully performed all 327 tasks (**Supplementary Data 4**). Removal of reactions from *iHsa* as part of the reconciliation process did not affect the completion of any metabolic tasks. Furthermore, the addition of new reactions to *iRno* and *iHsa* did not enable completion of 19 tasks explicitly intended to fail such as the *de novo* synthesis of essential amino. An important advantage of *iHsa* (and *iRno*) is that one unit of glucose regenerates 25.6 units of ATP with an unlimited supply of oxygen and 2 units of ATP in the absence of oxygen. Several new tasks were added that tested whether *iRno* and *iHsa* could use thermodynamically infeasible loops to regenerate ATP from ADP without a carbon-based energy or "fuel" source like glucose.

The presence or absence of all KEGG MODULEs were queried for rats and humans, revealing 2 human-specific modules and 1 rat-specific module. Each module described the ability of an organism to synthesize a product *de novo* from a starting substrate, as reported above for the rat-specific module M00129 where vitamin C can be synthesized from glucose. In KEGG, the human-specific modules for chenodeoxycholic acid synthesis from cholesterol and degradation of heparan sulfate into disaccharides were each characterized by a single missing enzyme in rats. For chenodeoxycholic acid synthesis, the blocked reaction, 3-alpha-hydroxysteroid dehydrogenase (EC 1.1.1.50) was not annotated for any rat enzymes. Upon review of experimental literature, the rat gene, *Akr1c14*, was reported to demonstrate this activity[10], suggesting that this module be reclassified as complete in both rats and humans. For heparan

sulfate degradation, manually assigning the rat enzyme, *Hgsnat*, to its known function (EC 2.3.1.78) was able to recapitulate this previously annotated human-specific function in rats[11].

Capturing the importance of L-gulonolactone oxidase in vitamin C synthesis

L-gulonolactone oxidase (*Gulo*) has been described as the critical enzyme for vitamin C synthesis that differentiates rats from humans[12] (**Supplementary Fig. 4a**). Using flux variability analysis (FVA)[13], 10 reactions were required by *iRno* to synthesize vitamin C under glucose minimal media conditions. Only one enzymatic reaction required for vitamin C synthesis in *iRno* that was also absent in *iHsa* was L-gulonolactone oxidase (EC 1.1.3.8) (**Supplementary Fig. 4b**). In agreement with the KEGG MODULE, “ascorbate biosynthesis, animals” (M00129), L-gulonolactone oxidase (K00103) was annotated as the only enzymatic step missing in humans (**Supplementary Fig. 4c**). Additionally, deleting *Gulo* blocked the ability of *iRno* to produce vitamin C, consistent with a *Gulo*-deficient strain of rat developed to study scurvy[14]. Artificially adding L-gulonolactone oxidase to *iHsa* would enable the human model to successfully complete the vitamin C synthesis task (**Supplementary Fig. 4d**), as previously described in a study that restored vitamin C synthesis in a human cell line using the murine ortholog of *Gulo*[15].

Vitamin C consumption was required for biomass synthesis in *iHsa* but not in *iRno*. The functional impact of vitamin C deficiency on cellular growth was simulated by constraining the uptake of the vitamin C exchange reaction to 1 fmol cell^{-1} hour^{-1} (physiological consumption rate[16]) or 0 (vitamin C deficiency) (**Supplementary Data 4**). The maximum theoretical flux through the biomass reaction of each model, containing equimolar amounts of vitamin C per cell (0.06 fmol cell^{-1}), was measured *in silico* using flux balance analysis[17]. When the uptake rate of vitamin C was decreased below 25% of normal physiological rates, the maximum possible growth rate was reduced exclusively in *iHsa* and not in *iRno*. Despite this distinction, limiting the uptake of vitamin C within an order of magnitude of the physiological uptake rate had no effect

on growth, suggesting that vitamin C is not likely a growth rate-limiting factor under normal conditions in either organism.

Defining physiological conditions and biomass compositions for human and rat hepatocytes

A novel system of reactions representing biomass synthesis was developed to enable cross-species predictions of growth between *iRno* and *iHsa* (**Supplementary Fig. 6**). New biomass metabolites were defined for each macromolecular subcomponent present in a hepatocyte including (percent of dry weight): DNA (2.3%), RNA (3.7%), lipids (17%), protein-incorporated amino acids (59%), free amino acids (3.7%), bile acids (.1%), and miscellaneous metabolites (11%) (**Supplementary Fig. 6a**). Miscellaneous metabolites included vitamins, and cofactors, and other metabolites present at high intracellular concentrations such as vitamin C, citrate, and glutathione. The relative abundances of individual metabolites within each subgroup were determined from several previously published studies [16,18-32]. Data directly comparing metabolite profiles between human and rat hepatocytes were available for amino acids and bile acids. To account for these differences within a generalized framework, species-specific reactions were added to *iRno* and *iHsa* for the synthesis of these two biomass metabolites (**Supplementary Fig. 6b**). For biomass components with similar compositions between rats and humans, shared reactions were used to produce estimated hepatocyte-specific compositions (**Supplementary Fig. 6c**). This new cross-species framework can be extended to formulate new biomass compositions for cross-species analyses within and between various cell or tissue types using the same centralized biomass precursor metabolites.

Physiological ranges for exchange reactions were determined using a consensus approach (**Supplementary Fig. 7**). Experimentally measured metabolite consumption and secretion rates were obtained for rat liver cells and rat hepatocytes from 6 existing studies[3,33-38]. Exchange

reaction equations were formulated such that negative and positive fluxes represented consumption and secretion, respectively. Flux measurements were standardized to units of fmol cell^{-1} hour^{-1} using previously described conversion rates[39]. In order to normalize quantitative measurements from different experimental systems, absolute flux measurements were median-scaled using metabolites measured in all 6 experiments to the average median value of the 3 *in vitro* experiments. To assign experimental observations as physiological constraints, lower and upper bounds for exchange reactions were determined based on minimum and maximum normalized values across all experimental observations.

Physiological constraints were applied to *iRno* and *iHsa* as either relaxed constraints for treatment-induced biomarker predictions (**Supplementary Fig. 7a**) or strict constraints for quantitative simulations of hepatocyte biomass (**Supplementary Fig. 7b**). Under relaxed and strict physiological constraints, lower bound values less than zero were used to allow nutrient uptake of measured metabolites (**Supplementary Fig. 7a**). Under strict physiological constraints, lower bound values greater than zero were also applied requiring secretion of urea, glucose, glutamate, aspartate, 3-hydroxybutyrate, and albumin. Upper bound values were also applied to require consumption or limit secretion of metabolites under strict physiological constraints (**Supplementary Fig. 7b**). Additionally, estimated uptake rates for 12 inorganic ions (**Supplementary Fig. 7d**) and 13 essential nutrients (**Supplementary Fig. 7e**) were assigned under both relaxed and strict physiological constraints. For metabolites with flux measurements available in rat and human hepatocytes, differences between species were considered negligible relative to feasible flux ranges with the exception of bile acids.

Under strict physiological conditions, species-specific constraints were formulated for the export of bile salts by hepatocytes (**Supplementary Fig. 7c**). In addition to the unique ability of rats to synthesize muricholic acids, the relative abundances of bile acids differed significantly in a

recent study that compared serum bile acid profiles of rats and humans[33]. Rat-specific and human-specific reactions were defined to produce an average bile salt measured for each organism, similar to species-specific reactions formulated for biomass synthesis. Under strict physiological conditions, a minimum flux of 0.4 fmol cell^{-1} hour^{-1} was required through a unified exchange reaction representing average bile salt production.

Supplementary References

1 Thiele, I. *et al.* A community-driven global reconstruction of human metabolism. *Nat Biotechnol* **31**, 419-425, doi:10.1038/nbt.2488 (2013).

2 Shlomi, T., Cabili, M. N. & Ruppin, E. Predicting metabolic biomarkers of human inborn errors of metabolism. *Mol Syst Biol* **5**, 263, doi:10.1038/msb.2009.22 (2009).

3 Mardinoglu, A. *et al.* Genome-scale metabolic modelling of hepatocytes reveals serine deficiency in patients with non-alcoholic fatty liver disease. *Nat Commun* **5**, 3083, doi:10.1038/ncomms4083 (2014).

4 Gabaldon, T. & Koonin, E. V. Functional and evolutionary implications of gene orthology. *Nature reviews. Genetics* **14**, 360-366, doi:10.1038/nrg3456 (2013).

5 Blazier, A. S. & Papin, J. A. Integration of expression data in genome-scale metabolic network reconstructions. *Front Physiol* **3**, 299, doi:10.3389/fphys.2012.00299 (2012).

6 Machado, D. & Herrgard, M. Systematic evaluation of methods for integration of transcriptomic data into constraint-based models of metabolism. *PLoS Comput Biol* **10**, e1003580, doi:10.1371/journal.pcbi.1003580 (2014).

7 Kanehisa, M. & Goto, S. KEGG: kyoto encyclopedia of genes and genomes. *Nucleic Acids Res* **28**, 27-30 (2000).

8 Kanehisa, M. *et al.* Data, information, knowledge and principle: back to metabolism in KEGG. *Nucleic Acids Res* **42**, D199-205, doi:10.1093/nar/gkt1076 (2014).

9 Gibbs, R. A. *et al.* Genome sequence of the Brown Norway rat yields insights into mammalian evolution. *Nature* **428**, 493-521, doi:10.1038/nature02426 (2004).

10 Penning, T. M., Jin, Y., Heredia, V. V. & Lewis, M. Structure-function relationships in 3alpha-hydroxysteroid dehydrogenases: a comparison of the rat and human isoforms. *J Steroid Biochem Mol Biol* **85**, 247-255 (2003).

11 Bame, K. J. & Rome, L. H. Acetyl-coenzyme A:alpha-glucosaminide N-acetyltransferase. Evidence for an active site histidine residue. *The Journal of biological chemistry* **261**, 10127-10132 (1986).

12 Kawai, T., Nishikimi, M., Ozawa, T. & Yagi, K. A missense mutation of L-gulono-gamma-lactone oxidase causes the inability of scurvy-prone osteogenic disorder rats to synthesize L-ascorbic acid. *The Journal of biological chemistry* **267**, 21973-21976 (1992).

13 Mahadevan, R. & Schilling, C. H. The effects of alternate optimal solutions in constraint-based genome-scale metabolic models. *Metab Eng* **5**, 264-276 (2003).

14 Mizushima, Y., Harauchi, T., Yoshizaki, T. & Makino, S. A rat mutant unable to synthesize vitamin C. *Experientia* **40**, 359-361 (1984).

15 Ha, M. N. *et al.* Functional rescue of vitamin C synthesis deficiency in human cells using adenoviral-based expression of murine l-gulono-gamma-lactone oxidase. *Genomics* **83**, 482-492, doi:10.1016/j.ygeno.2003.08.018 (2004).

16 Lykkesfeldt, J., Hagen, T. M., Vinarsky, V. & Ames, B. N. Age-associated decline in ascorbic acid concentration, recycling, and biosynthesis in rat hepatocytes--reversal with (R)-alpha-lipoic acid supplementation. *FASEB J* **12**, 1183-1189 (1998).

17 Varma, A. & Palsson, B. O. Stoichiometric flux balance models quantitatively predict growth and metabolic by-product secretion in wild-type Escherichia coli W3110. *Applied and environmental microbiology* **60**, 3724-3731 (1994).

18 Olsson, J. M., Eriksson, L. C. & Dallner, G. Lipid compositions of intracellular membranes isolated from rat liver nodules in Wistar rats. *Cancer Res* **51**, 3774-3780 (1991).

19 Yamada, M. *et al.* Biochemical characteristics of isolated rat liver stellate cells. *Hepatology* **7**, 1224-1229 (1987).

20 Bartles, J. R., Feracci, H. M., Stieger, B. & Hubbard, A. L. Biogenesis of the rat hepatocyte plasma membrane in vivo: comparison of the pathways taken by apical and basolateral proteins using subcellular fractionation. *J Cell Biol* **105**, 1241-1251 (1987).

21 Benga, G. & Ferdinand, W. Amino acid composition of rat and human liver microsomes in normal and pathological conditions. *Biosci Rep* **15**, 111-116 (1995).

22 Yang, L. Y., Kuksis, A., Myher, J. J. & Steiner, G. Origin of triacylglycerol moiety of plasma very low density lipoproteins in the rat: structural studies. *J Lipid Res* **36**, 125-136 (1995).

23 Gibbons, G. F., Khurana, R., Odwell, A. & Seelaender, M. C. Lipid balance in HepG2 cells: active synthesis and impaired mobilization. *J Lipid Res* **35**, 1801-1808 (1994).

24 Barle, H. *et al.* The concentrations of free amino acids in human liver tissue obtained during laparoscopic surgery. *Clin Physiol* **16**, 217-227 (1996).

25 Triguero, A. *et al.* Liver intracellular L-cysteine concentration is maintained after inhibition of the trans-sulfuration pathway by propargylglycine in rats. *Br J Nutr* **78**, 823-831 (1997).

26 Turunen, M., Olsson, J. & Dallner, G. Metabolism and function of coenzyme Q. *Biochim Biophys Acta* **1660**, 171-199 (2004).

27 Niklas, J., Noor, F. & Heinzle, E. Effects of drugs in subtoxic concentrations on the metabolic fluxes in human hepatoma cell line Hep G2. *Toxicol Appl Pharmacol* **240**, 327-336, doi:10.1016/j.taap.2009.07.005 (2009).

28 Zhao, M. *et al.* Vitamin B-6 restriction impairs fatty acid synthesis in cultured human hepatoma (HepG2) cells. *Am J Physiol Endocrinol Metab* **304**, E342-351, doi:10.1152/ajpendo.00359.2012 (2013).

29 Momchilova, A. *et al.* Resveratrol alters the lipid composition, metabolism and peroxide level in senescent rat hepatocytes. *Chem Biol Interact* **207**, 74-80, doi:10.1016/j.cbi.2013.10.016 (2014).

30 Qin, X. Y. *et al.* The effect of acyclic retinoid on the metabolomic profiles of hepatocytes and hepatocellular carcinoma cells. *PLoS One* **8**, e82860, doi:10.1371/journal.pone.0082860 (2013).

31 da Silva, V. R. *et al.* Targeted metabolomics and mathematical modeling demonstrate that vitamin B-6 restriction alters one-carbon metabolism in cultured HepG2 cells. *Am J Physiol Endocrinol Metab* **307**, E93-101, doi:10.1152/ajpendo.00697.2013 (2014).

32 Setchell, K. D. *et al.* Bile acid concentrations in human and rat liver tissue and in hepatocyte nuclei. *Gastroenterology* **112**, 226-235 (1997).

33 Garcia-Canaveras, J. C., Donato, M. T., Castell, J. V. & Lahoz, A. Targeted profiling of circulating and hepatic bile acids in human, mouse, and rat using a UPLC-MRM-MS-validated method. *J Lipid Res* **53**, 2231-2241, doi:10.1194/jlr.D028803 (2012).

34 Yang, H., Roth, C. M. & Ierapetritou, M. G. Analysis of amino acid supplementation effects on hepatocyte cultures using flux balance analysis. *OMICS* **15**, 449-460, doi:10.1089/omi.2010.0070 (2011).

35 Chan, C., Berthiaume, F., Lee, K. & Yarmush, M. L. Metabolic flux analysis of cultured hepatocytes exposed to plasma. *Biotechnol Bioeng* **81**, 33-49, doi:10.1002/bit.10453 (2003).

36 Lee, K., Berthiaume, F., Stephanopoulos, G. N. & Yarmush, M. L. Profiling of dynamic changes in hypermetabolic livers. *Biotechnol Bioeng* **83**, 400-415, doi:10.1002/bit.10682 (2003).

37 Banta, S., Yokoyama, T., Berthiaume, F. & Yarmush, M. L. Effects of dehydroepiandrosterone administration on rat hepatic metabolism following thermal injury. *J Surg Res* **127**, 93-105, doi:10.1016/j.jss.2005.01.001 (2005).

38 Banta, S. *et al.* Contribution of gene expression to metabolic fluxes in hypermetabolic livers induced through burn injury and cecal ligation and puncture in rats. *Biotechnol Bioeng* **97**, 118-137, doi:10.1002/bit.21200 (2007).

39 Sohlenius-Sternbeck, A. K. Determination of the hepatocellularity number for human, dog, rabbit, rat and mouse livers from protein concentration measurements. *Toxicol In Vitro* **20**, 1582-1586, doi:10.1016/j.tiv.2006.06.003 (2006).
